# Design and Utilization of a Direct Methanol Fuel Cell

**DOI:** 10.3390/membranes12121266

**Published:** 2022-12-14

**Authors:** Aser Alaa Ahmed, Malik Al Labadidi, Ahmed T. Hamada, Mehmet Fatih Orhan

**Affiliations:** Department of Mechanical Engineering, College of Engineering, American University of Sharjah, Sharjah P.O. Box 26666, United Arab Emirates

**Keywords:** fuel cell, direct methanol fuel cell, JENNY 600S DMFC, model, MATLAB, overall performance, multi-irreversibilities, operating temperature, output cell voltages

## Abstract

This study introduces a step-by-step, summarized overview of direct methanol fuel cell (DMFC) fundamentals, thermodynamic–electrochemical principles, and system evaluation factors. In addition, a parametric investigation of a JENNY 600S DMFC is conducted to simulate cell performance behavior under varying operating conditions. The system is mathematically modeled and solved in MATLAB and accounts for multi-irreversibilities such as the activation and ohmic and concentration overpotentials. The performance of the modeled system was validated against theoretical and experimental results from the literature. The results indicated that increasing the fuel cell’s operating temperature yields enhanced output cell voltages due to enhanced methanol oxidation reactions. Nevertheless, the maximum efficiency limits of the fuel cell tend to decrease with an increase in temperature. In addition, the model has also depicted that enhanced output cell voltages are associated with increased oxygen consumption, resulting in the lower exit flowrates of the reactants.

## 1. Introduction

**Figure 1:**
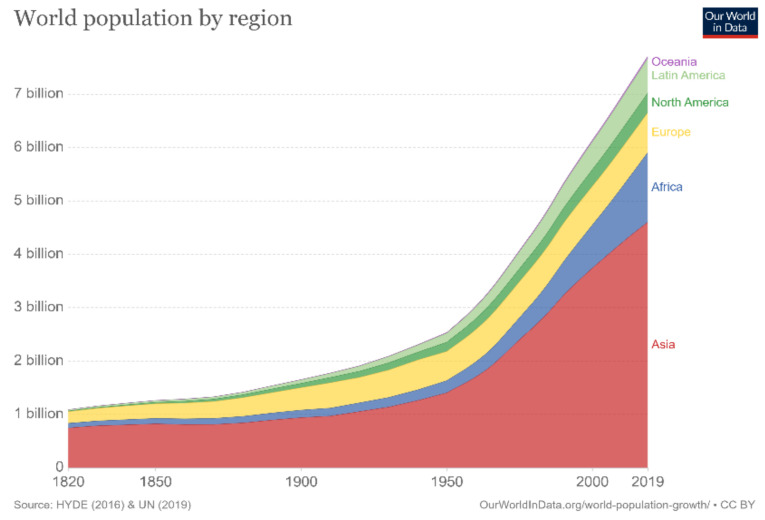
World population by region [1].

**Figure 2:**
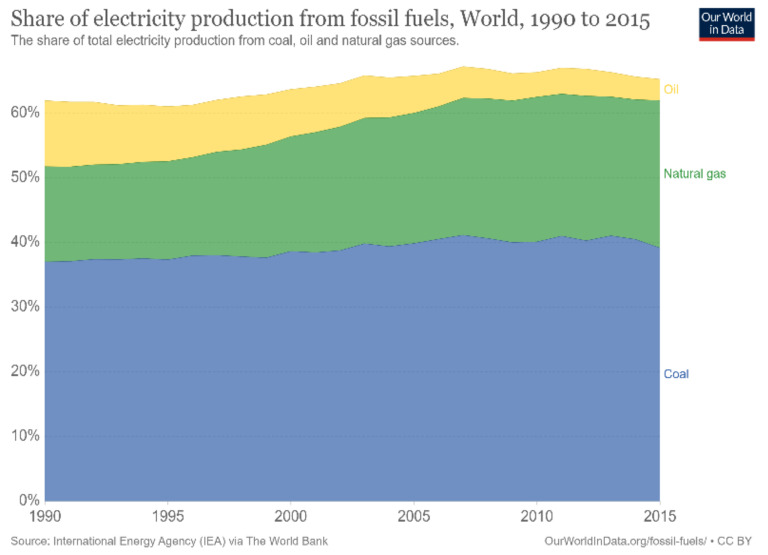
Share of electricity production from fossil fuels [2].

The world’s population has been increasing rapidly, and according to the United Nations, it is estimated to reach approximately 10 billion in the next 30 years, as depicted in Figure 1 [1]. This increase in population mandates accelerated technological advancements that can effectively and sufficiently meet all needs. Nevertheless, technological advancements require continuous and sustainable energy sources to facilitate their operations. Throughout the previous century, the world has relied heavily on fossil fuels to meet this demand. As evident in Figure 2 [2], more than 60% of the world’s electricity is supplied by burning fossil fuels. Coal is regarded as one of the most abundant fuel sources, providing approximately 40% of the total electricity production; followed by natural gas, contributing approximately 20%; and finally, oil, contributing approximately 5 to 10%. Notably, fossil fuels are considered nonrenewable and unclean sources of energy that can result in harmful emissions and greenhouse gas production. Greenhouse gases contribute to global warming, blocking longwave radiation within the Earth’s atmosphere, thus increasing the planet’s temperature. In the long run, this increase in temperature may have adverse effects on the global environment; for instance, the elevated temperatures can accelerate the melting process of vast amounts of ice at the poles, which would, consequently, lead to rising sea levels, floods, and other natural disasters. One way of tackling the aforementioned problems is to develop new ways of generating clean energy that ensure sustainable development. Nowadays, most of these developments utilize renewable energy sources, such as solar energy, wind energy, and hydropower. However, these types of energy sources are mostly intermittent and introduce a spatial and temporal gap between the energy’s availability and its consumption by end-users. Overcoming this hurdle mandates the development of suitable energy systems for the grid that increase the complexity of operation [3].

Consequently, this brought rise to electrochemical generators, such as fuel cells, for clean energy generation, as they are regarded as a better and more feasible alternative. Sir William Robert Grove, a Welsh inventor, physicist, and patent lawyer, hypothesized a reverse electrolysis process that could be used to generate electrical power. In 1893, his hypothesis was deemed successful upon building an electrochemical device known as a fuel cell. It is a green technology where hydrogen (fuel) is supplied from the anode side to a catalyst layer where it decomposes to form protons and electrons. The protons migrate to the cathode side through a proton exchange membrane, which serves as an electrolyte that acts as a barrier between two electrodes and an ion-conductive medium [4]. The electrons, on the other hand, migrate to the cathode through an external circuit, thus creating a flow of electrical current that can be utilized to power various applications. The protons react with the supplied oxygen and electrons at the cathode to form water and heat, these being the only side products [5]. Such types of fuel cells are referred to as proton exchange membrane fuel cells (PEMFCs). Nowadays, the main competitors to fuel cells are solid-state and flow batteries. Both batteries and fuel cells are quite similar in the sense that they are electrochemical cells that utilize an electrolyte that is confined between two electrodes. In addition, both use an internal oxidation–reduction reaction to convert the fuel’s chemical energy to electrical energy. The main difference, however, is that the electrodes of a battery are typically metals, whereas in fuel cells they mainly consist of ion-conducting media, carbon-supported catalysts, and electron-conducting fibers [6]. A solid-state battery uses the chemical energy in electrodes to fuel the electrochemical reaction. Flow batteries, however, utilize the chemical energy provided by two chemical components dissolved in liquids that are pumped past a membrane held between two electrodes. They offer mutually independent output power and capacity that rely on the size of the stack’s electrolyte volume and concentration [7]. Compared with conventional solid-state batteries, they offer higher energy densities and enhanced life cycles. Nevertheless, compared with a fuel cell, their life cycle, regardless of the type of battery, is limited, and they only operate as long as the electrodes’ material has not yet depleted. Fuel cells, on the contrary, continuously operate as long as there is a continual supply of reactants. In addition, batteries suffer from numerous technical issues that limit their applications, such as their low energy density, limited charge/discharge cycle, aging, and their potentially hazardous/explosive nature during battery failure [8,9]. In the case of fuel cells, the aforementioned problems are not an issue. In fact, no leakage or corrosion of fuel cell components takes place when it is not operated; therefore, they do not age/deteriorate with time, unlike batteries [6]. Nevertheless, the operational costs of fuel cells are one of the major issues hindering their widespread commercialization, hence the extensive research efforts being conducted within this realm [10].

One of the most crucial components of any fuel cell is the membrane electrode assembly (MEA), which includes a polymer membrane, electrodes (anode and cathode), a gas diffusion layer (GDL), and a catalyst layer (CL). Considerable research efforts are devoted to the development of MEAs because they are highly susceptible to degradation. In addition, electrolytes and bipolar plates are also important components of a fuel cell. Each of these components performs a different role, and together, they facilitate the generation of electrical power. Most fuel cells are maintenance-free due to the simplicity of the fuel cell structure and their long-lasting components, such as the electrodes, which are not consumed during the electrochemical reactions that occur. There are numerous types of fuel cells, as evident in Table 1, and all can come in different shapes and sizes. The larger the size/the bigger the fuel cell stack, the higher the electrical power production of the system. Consequently, this enables the widespread utilization of fuel cells within a multitude of applications. For instance, they can be utilized for emergency backup supplies, remote-area power supplies, and combined-heat-and-power generation. They can also be utilized as portable power generators for a diverse range of electronic devices (e.g., laptops, cell phones, radios, etc.). The transportation sector has also initiated the integration of fuel cell systems within their applications. For instance, they are widely used as onboard auxiliary power units in various automotive types [11]. They are also commonly used to operate light traction vehicles (e.g., scooters, personal wheelchairs, airport tugs, etc.) [6] and light-duty electric vehicles (e.g., Hyundai’s ix35).

**Figure 3:**
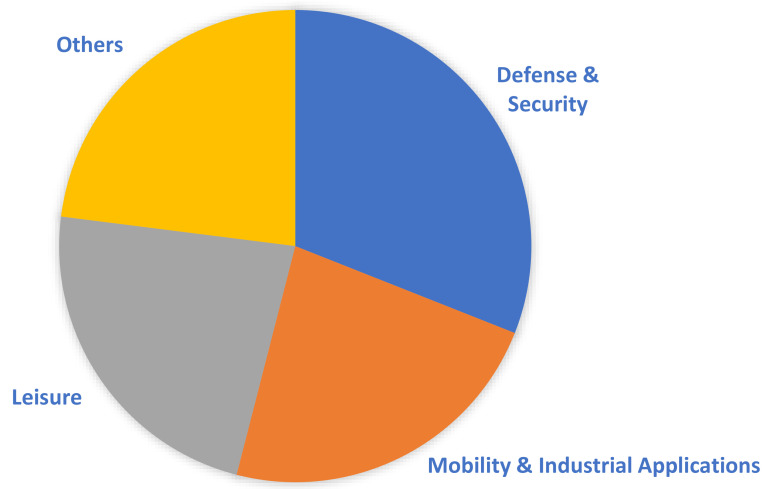
Main applications of DMFCs.

A direct methanol fuel cell (DMFC) is a type of fuel cell that uses liquid methanol (CH_3_OH) as fuel and a proton exchange membrane as the electrolyte. Currently, a significant portion of the global DMFC market is controlled by four major companies: SFC Energy AG, Oorja Corporation, Fujikura Ltd., and Siqens. As evident in Figure 3, the DMFC market mainly revolves around mobility and leisure applications, as they can reach a wider consumer base. Nevertheless, the military defense and security sectors of numerous countries constitute a significant portion of the market, integrating DMFC systems within their applications. In general, portable military equipment that is fuel-cell-powered mandates certain standards; namely, the power source should be compact and lightweight, and the fuel supply, water management, and heat dissipation should be controlled and well regulated [12]. This is facilitated by the use of DMFCs because they eliminate requirements for fuel reforming and/or large onboard hydrogen storage tanks that are essential for the operation of PEMFC systems, are eco-friendly, and are highly efficient. Therefore, this enables the fabrication of a simpler design that can potentially have low-volume and lightweight packaging. Furthermore, liquid methanol can be easily supplied from an external container or in small cartridges based on demand [13]. The utilization of methanol as a fuel is advantageous for portable use, especially within military applications, e.g., soldier power (<500 W), sensor power (0–100 W), and auxiliary power units (0.5–10 kW) [13] because hydrocarbon-based liquid fuels, in general, have high energy density. Methanol, for instance, has an energy density of approximately 6 kWh/kg, which is, relatively, much higher than other commercialized batteries [8].

Even though portable DMFC systems have been previously investigated in the literature, the novelty of this study relies on constructing a comprehensive and effective case study that investigates the performance of DMFCs in military-based applications by analyzing a JENNY 600S DMFC system manufactured by SFC Energy AG in Brunnthal/Munich, Germany. This analysis is conducted by mathematically modeling the DMFC system using appropriate electrochemical equations in the MATLAB environment. The study explored the system’s power density, maximum efficiency, and irreversibilities and the consumption rates of methanol and oxygen.

## 2. Fundamental Principles of Direct Methanol Fuel Cells

**Figure 4:**
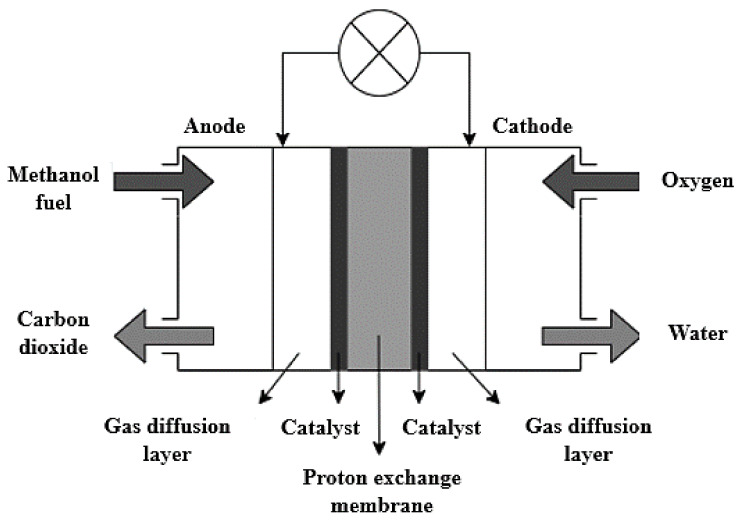
Schematic diagram of the working principle of a DMFC.

The chemical reactions taking place within a DMFC system are highlighted in Equations (1)–(3). In general, the methanol fuel is oxidized in the presence of water at the anode catalyst layer, thus releasing electrons and protons during this process, as shown in Equation (1). The electrons then migrate through an external circuit to the cathode, whereas the protons are transported through the electrolyte membrane to the cathode. The protons and electrons then react with the oxygen supplied at the cathode catalyst layer to produce water, as shown in Equation (2). Figure 4 highlights the general structure of a DMFC.

Anode:

(1) $${CH}_{3}\mathrm{OH}+H_{2}O\to {\mathrm{CO}}_{2}+6H^{+}+6e^{-}$$

Cathode:

(2) $$\frac{3}{2}O_{2}+6H^{+}+6e^{-}\to 3H_{2}O$$

Overall reaction:

(3) $${\mathrm{CH}}_{3}\mathrm{OH}+\frac{3}{2}O_{2}\to {\mathrm{CO}}_{2}+2H_{2}O$$

DMFC systems can be categorized as active, semi-passive, or passive depending upon the supply mode of the fuel and the oxidant. Active DMFCs, for instance, make use of pumps, sensors, heaters, fans, and various humidity components to facilitate the accurate control of the fuel and oxygen supply, thus producing the highest performance. Passive DMFCs, on the other hand, do not rely on auxiliary components for the supply of fuel/oxidant but instead depend on direct transportation from methanol tanks and ambient surroundings to the membrane electrode assembly with the aid of gravity and concentration gradients [14]. This category of DMFC system is more compact and reliable, thus making it a suitable candidate for portable applications. Finally, semi-passive DMFCs utilize passive anodes and active cathodes or vice versa.

DMFCs, as previously mentioned, have several advantages over rechargeable batteries. Nevertheless, their commercialization has been hindered due to a few technical limitations. Methanol crossover is one major limitation that such fuel cells suffer from, which has ignited widespread attention in terms of the implemented research efforts to tackle this problem. This is a phenomenon that occurs due to the inherent water transport property of the Nafion membrane (manufactured by The Chemours Company in the USA) and the miscibility between methanol and water [14]. This causes a chemical reaction to take place between the crossed-over methanol and oxygen at the cathode, thus reducing the cell voltage and deteriorating the fuel efficiency since it is not involved in generating electricity. Water management is another challenging aspect that needs to be accounted for during DMFC operations. In general, it is essential to supply a water-to-methanol ratio to the anode catalyst layer that exceeds 1:1 to avoid the partial oxidization of methanol into formic acid, methylformate, and formaldehyde [15], which can possibly poison the catalyst layer and impede its performance. In addition, sufficient water is required to ensure the proper hydration of the electrolyte membrane because the proton-conductive resistance of Nafion, for instance, is inversely proportional to its water content [16]. Furthermore, as evident in Equation (1), a byproduct of a DMFC is carbon dioxide (CO_2_). Even though it is regarded as a very stable gas and is essential for the survival of most living organisms and cycles within the ecosystem, it can accumulate in the atmosphere for long periods of time. Indeed, the increasing amount of fossil fuel being incinerated within factories, automobiles, etc., has accelerated the rate of CO_2_ emissions beyond acceptable natural levels, thus adversely affecting the global environment, as previously discussed. In addition to environmental concerns, the cell’s CO_2_ generation can have undesirable effects on the operation of a DMFC and hinder its performance. In other words, it introduces the risk of CO_2_ crossover through the electrolyte membrane due to pressure gradients between both electrodes. It has been proven that this crossover rate can increase with an increased current density due to higher CO_2_ production [14]. This phenomenon occurs at a higher rate when methanol concentrations are high, as it increases the mass transport resistance and makes CO_2_ release from the anode side more difficult, thus encouraging its crossover to the cathode and escaping into the environment. To overcome this hurdle, fuel cell systems can be equipped with CO_2_-capturing mechanisms. Such mechanisms allow for the separation of CO_2_ from gas streams, which also facilitates its purification for subsequent use [17].

## 3. Background

**Figure 5:**
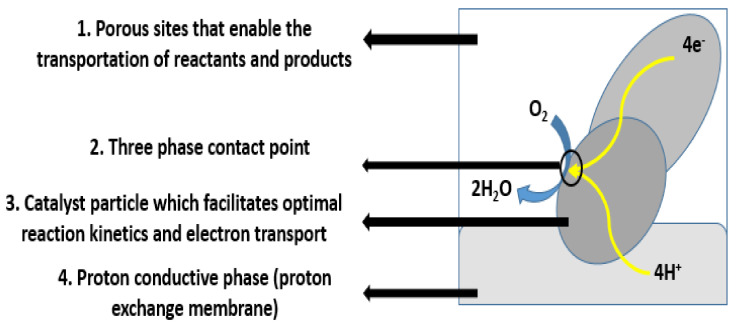
Schematic representation of the three-phase contact boundary for the cathode’s catalyst layer in a DMFC adapted from [21].

The performance of any fuel cell highly relies on the type and performance of the various components that constitute the technology. For this reason, this section aims to provide a brief overview of the various types of components and materials of a typical DMFC. Electrodes, for instance, are essential components in any type of fuel cell. Electrodes are mainly made of porous carbon fibers. In general, carbon-based materials are commonly utilized as electrode materials owing to their long life cycles, high electrical conductivity, chemical stability, cost, and ability to operate under oxidizing media [18]. In addition, they are also capable of providing a larger specific area and better 3D network structures, which facilitate enhanced electrochemical reactions. To catalyze reactions, platinum or precious-metal-based catalysts are added to the porous carbon fiber mixture, forming a catalyst layer. The electrodes must also enable reactants to reach the catalyst layers and reaction products to exit. The contact point of the reactants, catalyst, and electrolyte is conventionally referred to as the three-phase interface, as shown in Figure 5. To achieve acceptable reaction rates, the effective area of active catalyst sites must be several times higher than the geometric area of the electrode [19]. Therefore, electrodes are made porous by forming three-dimensional networks, and the three-phase interfaces are located within these networks. In addition, selecting the appropriate type and amount of catalyst within fuel cell applications is imperative, as they play a major role in determining the cell’s reaction rate and performance. Consequently, numerous catalyst types have been investigated. One of the most commonly resorted to catalysts is platinum (Pt). In general, Pt is expensive, and its stability is very poor and needs to be stabilized by an inert support. In addition, Pt by itself mitigates serious aggregation and coarsening during the catalytic reaction process, which reduces the electrode lifetime [20]. For this reason, Pt is commonly supported on a carbon black matric (Pt/C). Doing so facilitates enhanced reaction kinetics with minimized Pt loadings, thus reducing costs. Nevertheless, the intermediate CO formed during the methanol oxidation reaction strongly adsorbs into the Pt catalysts, which reduces the number of available reaction sites (three-phase interface) and hinders the catalytic activity of Pt and the overall cell performance. In an attempt of overcoming this problem, studies have investigated the impacts associated with alloying Pt with other transition metals. To date, nearly all transition metals—such as ruthenium (Ru), rhodium (Rh), palladium (Pd), osmium (Os), iridium (Ir), tin (Sn), nickel (Ni), copper (Cu), etc.—have been alloyed with Pt and tested for activity as methanol oxidation reaction (MOR) catalysts. Ru has been shown to be the most effective supplementary element. Despite the advantages associated with PtRu catalysts, their cost is still too high for commercialization within DMFC applications due to the relatively high cost of Ru. It is also worth noting that reports on non-Pt catalysts are much less common than those on Pt and Pt-based catalysts. To the best of our knowledge, there exist few active materials for methanol electro-oxidation, such as tungsten carbide (WC), cobalt–copper (CoCu) alloy, nickel cobaltite (NiCo_2_O_4_), zinc oxide (ZnO), and palladium–tin (IV) oxide composites (Pd/SnO_2_). Nevertheless, these catalysts are active for MORs only within alkaline conditions [20].

Recently, nanostructured carbons, including carbon nanotubes, carbon nanofibers, and ordered mesoporous carbons (OMC), have been exploited as new carbon support structures for DMFC catalysts to enhance their catalytic activity. Among the various nanostructured carbon supports, OMCs are inducing widespread attention as support materials for DMFCs due to their high surface area, uniform mesopores, and high thermal and chemical stabilities [20]. Apart from the aforementioned catalysts, Table 2 compares various other Pt-based catalysts from different studies based on a set of performance metrics.

Apart from a catalyst’s activity, its stability, durability, and cost are also imperative to account for. The synthesis process of the catalyst system should be simplified with inexpensive agents and suitable methods for mass production. Recent advances in catalysts show significantly improved MOR activity and durability, which has increased the potential commercialization of DMFC applications [22]. Despite the bright prospects of DMFCs, numerous technical challenges are yet to be tackled. For instance, the dissolution of transition metals during actual fuel cell operations leads to performance degradations. Moreover, the thick catalyst layer in the MEA influences gas diffusion and proton transfer, which leads to a lower utilization efficiency because the transition metals can hardly oxidize methanol.

Fuel cell efficiencies and power densities strongly depend on the conductivity of the employed electrolyte. DMFC applications mandate the utilization of acidic electrolytes to aid in the rejection of carbon dioxide. The electrolyte should acquire three main properties in a DMFC, which are as follows [22]:

It should be stable under the cell’s operating conditions;
It should possess high proton conductivity;
It should have low permeability for methanol.

A proper choice of electrolyte can reduce methanol crossover, enhance electrochemical reactions, and lower the cell’s overall cost. It also plays a major role in determining the fuel permeation rate and choice of catalyst. Proton-conducting electrolytes have been preferred over alkaline electrolytes for several decades for practical reasons, e.g., to avoid carbonation and corrosion and acquire better electrolyte management [23]. In addition, the conductivity of PEM-based electrolytes is higher owing to the higher diffusion coefficient of protons compared with the hydroxide ions in alkaline electrolytes [24]. The standard electrolyte membrane of DMFCs is usually a perfluoro sulfonic acid membrane, such as Nafion, which is also widely used in PEMFCs [25]. It is widely known for its excellent chemical, electrochemical, and high proton conductivity properties, which are derived from its unique chemical structure. An alternative to Nafion includes non-fluoropolymer membranes with functional groups (e.g., sulfonic acid groups or quaternary ammonium groups), which can be cheaper than the classic perfluoro sulfonic membranes used in PEMFCs; in some cases, they are also characterized by less methanol crossover. However, the lifetime and durability of the aforementioned electrolytes require additional investigations. Various other Nafion-based electrolytes are compared in Table 3, highlighting the performance of the cell that employs them. Liquid electrolytes have also been used in half-cell studies in an attempt to mimic the behavior of solid-state electrolytes within single cells and stacks. These types of electrolytes can be categorized as the following [25]:

Inorganic acids, such as H_2_SO_4_, HClO_4_, and H_3_PO_4_;
Superacids, such as trifluoromethanesulfonic acid (TFMSA);
Buffers, such as CO_3_^2−^/HCO_3_^−^;
Alkaline electrolytes, such as KOH.

However, due to extremely high methanol crossovers, this concept has been abandoned in favor of using Nafion.

Significant progress in electrolyte development for DMFCs has been made; nevertheless, it is difficult at present to identify the best membrane. This is because the choice of a proper membrane stems from several considerations, including device application, range of operating conditions, and costs. One commercial membrane that offers a suitable compromise among the various requested properties is produced by Polyfuel [22]. The Polyfuel membrane is especially suited for passive DMFCs. Its performance is comparable and even superior to that of Nafion-based devices, with its lifetime exceeding 5000 h. Although its stability is not comparable to that of Nafion (60,000 h in PEMFCs), it is deemed a suitable candidate for portable applications.

In a DMFC, bipolar plates separate the reactant gases from adjacent cells, electrically connect the cells, and act as a support structure. The bipolar plates have reactant flow channels on both sides, forming the anode and cathode compartments of unit cells on opposite sides of the bipolar plate. Most bipolar plates are made of stainless steel or graphite. Stainless steel plates are heavy components for portable power systems. Solid graphite plates are highly conductive, chemically inert, and resistant to corrosion; however, they are expensive and costly to manufacture [19]. Flow channels are machined or electrochemically etched in graphite or stainless-steel bipolar plate surfaces. Nevertheless, the discussed bipolar plates are not suitable for mass production, and, therefore, new types of bipolar plates are required. This, consequently, led to the rise of polymer composites because of their relatively low cost, weight, and ease of manufacturing.

The GDL enables the reactant gas to diffuse its way to the active layer (the catalyst layer supported on porous carbon fibers). They are mainly fabricated from pure porous polytetrafluoroethylene (PTFE), where PTFE suspensions are mixed with sugar or ammonium carbonate. The mixture is then heated at elevated temperatures to induce bubbles that result in the formation of porous PTFE films [26]. It is imperative for GDLs to be hydrophobic and electrically conductive. Nevertheless, both properties cannot be simultaneously achieved, and a tradeoff must exist. This is because enhancing the electrical conductivity of the GDL mandates mixing high concentrations of carbon black. Increased concentrations of carbon black, however, result in a loss of hydrophobicity.

**Figure 6:**
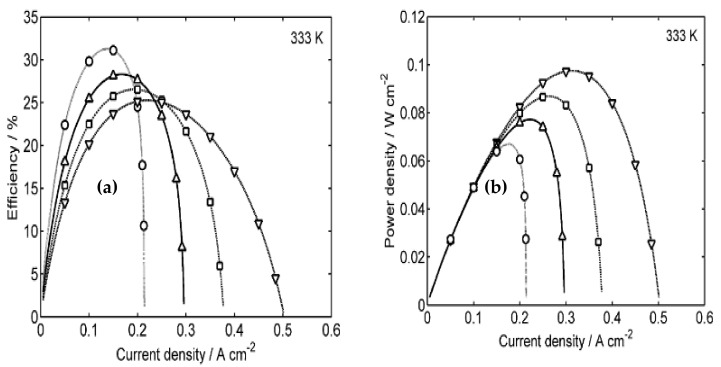
(**a**) Efficiency and (**b**) power density vs. current density plots at an operating temperature of 333 K and varying methanol concentrations. (◦) 0.75 M, (∆) 1.0 M, (□) 1.25 M, and (∇) 1.5 M [27].

**Figure 7:**
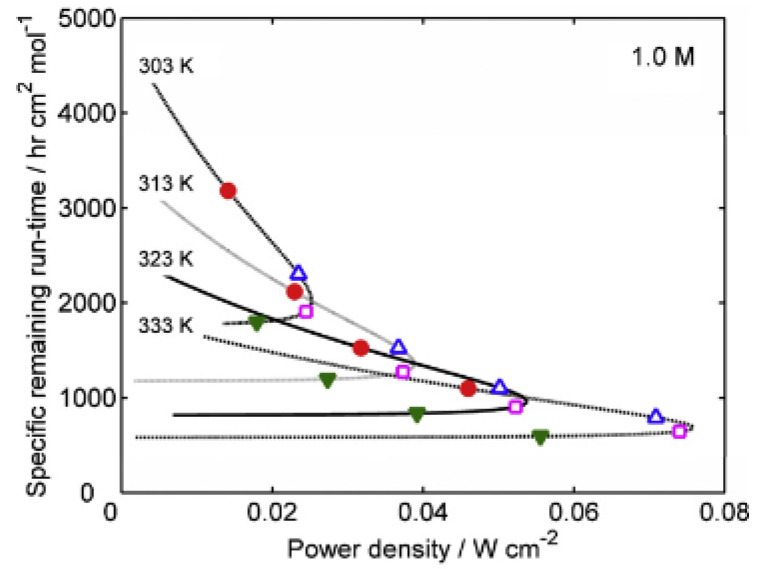
The specific remaining runtime under various operating temperatures and a fuel concentration of 1.0 M. (●) 0.5 V, (∆) 0.4 V, (□) 0.3 V, and (∇) 0.2 V [27].

In order to investigate the impacts associated with all the aforementioned fuel cell components and be able to further develop them, characterizing the performance of a fuel cell that employs them is imperative, as it shall serve as an indicator of the feasibility of the different components. Power efficiency is a major performance metric that quantifies the performance of any fuel cell based on its electrical power output. Determining this performance metric within DMFC applications is challenging due to fuel crossover and the complex cell architecture of fuel circulation. Notably, a semiempirical model has been developed to evaluate the efficiency of a DMFC under various conditions [27]. In that study, the power density and efficiency were presented as explicit functions of the operating temperature, fuel concentration, and current density and were compared to the experimental results, as shown in Figure 6. In addition, the leakage current effects were also analyzed with a mathematical model for transient leakage current and methanol concentration [28]. The remaining runtime of a DMFC was also estimated and compared to actual time periods obtained experimentally, as shown in Figure 7. In addition, a novel composite anode layer for the DMFC was composed of a dual-layer anode based on a catalyst-coated membrane technique. The inner sublayer, with a dense morphology, effectively suppressed methanol crossover, whereas the outer sublayer enhanced the electrochemical surface area and increased catalyst utilization [29]. This anodic improvement resulted in a 40% increase in power density. Moreover, the effects of crossover were assessed by analyzing the content of methanol in the cell exhaust, resulting in a method of reducing crossover. It was shown that the energy efficiency of a DMFC was dependent on the cell voltage, the crossover of methanol and oxygen, and the average number of electrons released per methanol molecule at the anode [28]. The results showed that errors due to crossover were insignificant when currents were limited by mass transport, while crossover must be considered at lower potentials. Furthermore, a quantitative and qualitative analysis of the reliability of a DMFC system (balance of plant) was conducted. The qualitative method used fault tree analyses to examine four main causes of total system failure [30]. The two key components of the reliability data were the failure rate and mean time before failure for the equipment, which provided a mathematical measure for the probability of failure. In another study, the contributions of different types of irreversibilities, including the overpotentials at the anode and cathode; methanol crossover; contact resistance; and proton conductivity of the membrane, were investigated. The results showed that the overpotentials, in conjunction with methanol crossover, were considered the major exergy destruction sources inside the cell [31]. It was noted that exergy losses due to electrochemical reactions were more significant at higher current densities. In contrast, exergy destruction by methanol crossover at the cathode played a more important role at lower currents. In addition, a two-dimensional, two-phase, passive alkaline anion exchange membrane direct methanol fuel cell (AAEM-DMFC) model was developed to understand the role of a microporous layer (MPL) and the effect of porous media wettability on species transport [32]. The results showed that different regions of the polarization curve exhibited different dependencies on the methanol feed concentration. Eventually, for good water distribution and low methanol crossover, porous layers with the desired properties should be designed, and simulation results may help optimize water management and improve methanol crossover issues.

## 4. System Description

This case study is based on a DMFC manufactured by the SFC Energy Company. In general, the products of the company serve a wide range of applications within clean Energy and mobility, defense and security, and the oil and gas sectors. The investigated DMFC is commonly referred to as JENNY 600S. It utilizes liquid methanol as a fuel and weighs 1.7 kg. Consequently, the developed fuel cell system facilitates around 80% weight reduction as it limits the number of spare batteries that would have otherwise been required to power electrical-based applications. In addition, the system produces a nominal power of 25 W and has high energy with the capability of operating for up to 72 h with the use of only 3–4 350 mL cartridges. Table 4 further presents the technical data of the DMFC system, which are utilized in the mathematical formulations highlighted in Section 5 to analyze its performance in the MATLAB environment.

## 5. Analysis

Using the technical data shown in Table 4, the gravimetric power density was calculated by dividing the nominal power by the system weight. In our case, the total system weight accounts for the mass of the fuel cell and the mass of a single loaded cartridge.

(4) $$P_{e,g}=\frac{P_{e}\;}{W}$$

The volumetric power density, on the other hand, can be obtained by dividing the fuel cell’s output power by the system’s total volume.

(5) $$P_{e,v}=\frac{P_{e}\;}{V}$$

Although the gravimetric and volumetric power densities are simple to calculate, they are also very descriptive. Thus, these two characteristics are normally used for power comparisons between systems.

To study any type of fuel cell, Gibbs free energy calculations are essential. Gibbs free energy can be defined as the energy available to perform external work, neglecting any work performed by changes in pressure and/or volume. Since work performed by a change in volume/pressure between the input and output is not captured by the fuel cell, Gibbs free energy is ideal for fuel cell analysis. The change in the Gibbs free energy of the formation provides us with the required energy released from the system. This change is the difference between the Gibbs free energy of the products and reactants.

Using the net DMFC reaction,

(6) $$CH_{3}OH+\frac{3}{2}O_{2}\to 2H_{2}O+CO_{2}$$

The molar enthalpy ${\bar{h}}_{f}$ of formation is

(7) $$2{\left({\bar{h}}_{f}\right)}_{H_{2}O}+{\left({\bar{h}}_{f}\right)}_{CO_{2}}-{\left({\bar{h}}_{f}\right)}_{m}-\frac{3}{2}{\left({\bar{h}}_{f}\right)}_{O_{2}}$$

The molar entropy $\bar{S}$ of formation is

(8) $$2{\left(\bar{S}\right)}_{H_{2}O}+{\left(\bar{S}\right)}_{CO_{2}}-{\left(\bar{S}\right)}_{m}-\frac{3}{2}{\left(\bar{S}\right)}_{O_{2}}$$

The molar Gibbs free energy of formation $∆{\bar{g}}_{f}$ is

(9) $$∆{\bar{g}}_{f}=∆{\bar{h}}_{f}-T∆\bar{S}$$

where *T* is the operating temperature of the DMFC.

In fuel cells, if there are no losses in the fuel cell or if the process is reversible, then all the Gibbs free energy is converted into electrical energy. Therefore, the Gibbs free energy values are used to calculate the reversible theoretical voltage of the fuel cell, as shown below:

(10) $$E=-\frac{∆{\bar{g}}_{f}}{𝓏F},$$

where *F* is the Faraday constant, and *z* is the number of electrons per mole of fuel. In DMFCs, *z* is equal to six since one mol of methanol fuel provides six electrons.

Since fuels are usually burned to produce energy, the efficiency can be determined by comparing the electrical energy produced from the fuel cell with the heat that would be produced by normally burning the fuel. The enthalpy of combustion is taken to be the same as the change in the enthalpy of formation that was previously calculated. However, the energy produced by the fuel cell can be effectively taken from the change in the Gibbs free energy. The maximum efficiency limit is also known as the thermodynamic efficiency and can be calculated as shown below:

(11) $$\;\eta =\frac{∆{\bar{g}}_{f}}{∆{\bar{h}}_{f}}\times 100\%$$

One way of characterizing the performance of any fuel cell is to measure the inlet and exit flowrates of the oxidizing agent, fuel, and water and their rate of consumption within the system. The equations represented below aid with calculating the aforementioned parameters. To facilitate the formulations of the mass flowrates, it is imperative to calculate the output voltage ($V_{c}$) of the fuel cell system:

(12) $$V_{c}=\frac{P_{e}}{n\times I},$$

where *I* is the operatic electric current of the fuel cell, and *n* is the number of cells within the DMFC system. It is important to note that the JENNY 600 DMFC system utilizes 32 cells.

That being said, the O_2_ consumption rate can be calculated as follows:

(13) $${\dot{m}}_{o_{2},c}=\frac{0.032P_{e}}{4FV_{c}}.$$

In addition, the rate of air consumption and supply can be calculated using Equations (14) and (15), respectively.

(14) $${\dot{m}}_{air,c}=\frac{0.02897P_{e}}{4FV_{c}},$$

(15) $${\dot{m}}_{air,s}={\dot{m}}_{air,c}\lambda$$

where λ is the stoichiometric value and is treated as one throughout the analysis conducted within the study. The exit mass flowrate of air, on the other hand, can be computed as follows:

(16) $${\dot{m}}_{air,e}={\dot{m}}_{air,s}-{\dot{m}}_{o_{2},c}.$$

The rate of methanol consumption and water production can be calculated using Equations (17) and (18), respectively.

(17) $${\dot{m}}_{methanol,c}=\frac{0.03204P_{e}}{6FV_{c}},$$

(18) $${\dot{m}}_{H_{2}O,p}=\frac{0.01802P_{e}}{2FV_{c}}.$$

Apart from the aforementioned flowrates, polarization curves are another effective way of characterizing the performance of a fuel cell by gaining insight into its voltage losses. These losses can be classified as activation losses, crossover losses, ohmic losses, and concentration losses. The activation losses are accounted for by utilizing Tafel’s equation [33].

The equation below enables the calculation of the actual cell voltage, which accounts for all of the aforementioned voltage drops.

(19) $$V_{c,actual}=E_{oc}-E_{activation}-E_{ohmic}-E_{concentration.}$$

where $E_{activation}$ is calculated as follows:

(20) $$E_{activation}=E_{anode}-E_{cathode.}$$

$E_{oc}$ is the reversible open circuit voltage, $E_{activation}$ is a voltage that accounts for both activation and crossover losses, $E_{ohmic}$ is the voltage for ohmic or resistive losses, $E_{concentration}$ is the voltage losses from mass transport or changes in concentration, and $E_{anode}$ and $E_{cathode}$ are the activation and crossover losses at the anode and cathode, respectively.

## 6. Results and Discussion

This section commences by highlighting a few general performance characteristics of the investigated DMFC system and then proceeds to highlight the various irreversibilities that impede the performance of the fuel cell and how the performance of the fuel cell is impacted by varying its operating temperature.

**Figure 8:**
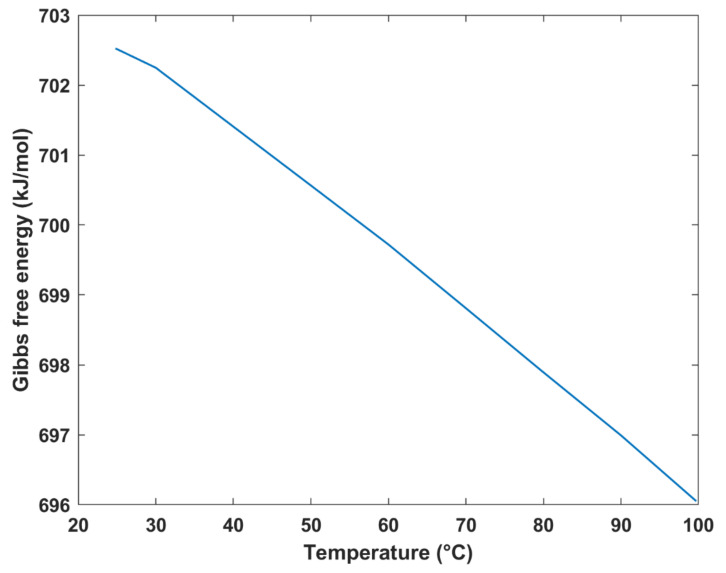
Gibbs free energy vs. temperature.

The variation of Gibbs free energy with temperature is plotted in Figure 8. As evident, as the temperature increases, the Gibbs free energy decreases. The reason for the decrease in Gibbs free energy is attributed to the increase in entropy as a result of the increasing temperature. The obtained results were consistent with those obtained in different studies. For instance, a study involving an analytical model of a DMFC showed that the Gibbs free energy was 702.7 kJ/mol at a temperature of 25 °C [34], which was consistent with the value obtained by this study. It is important to note that a temperature range of 25–100 °C was selected to avoid the cell drying up.

**Figure 9:**
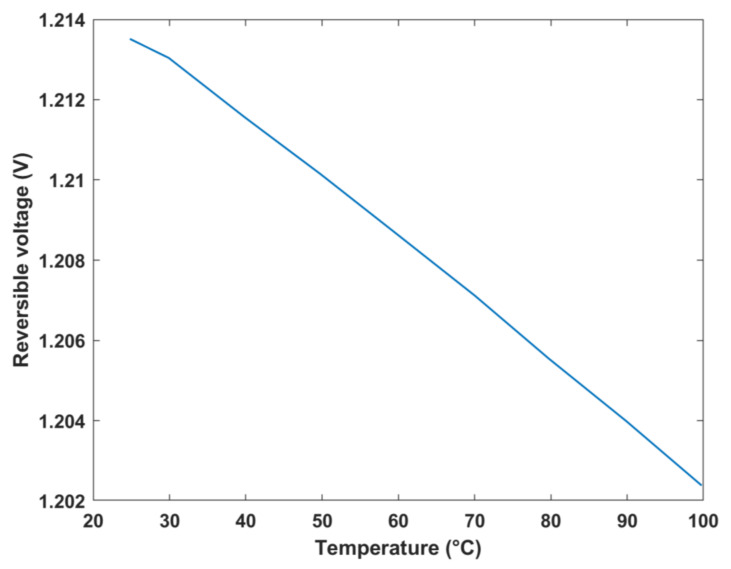
Reversible voltage vs. temperature.

**Figure 10:**
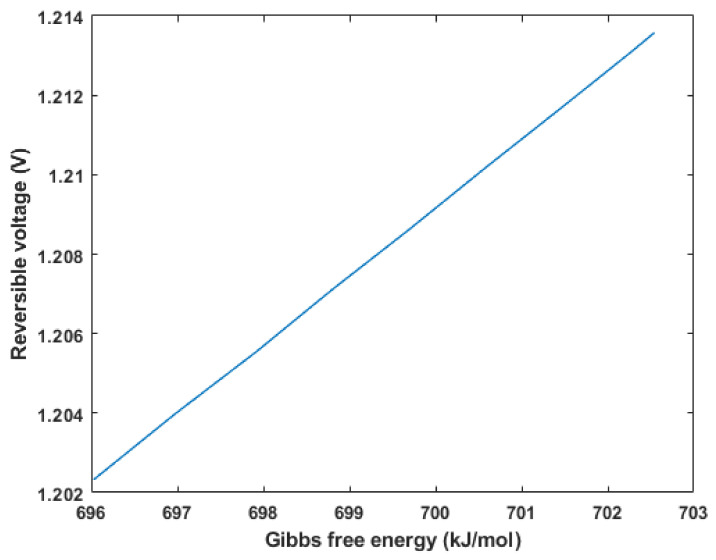
Reversible voltage vs. Gibbs free energy.

The reversible cell voltage was plotted against temperature and Gibbs free energy change in formation, as shown in Figure 9 and Figure 10, respectively. The results depict that the maximum reversible cell voltage obtained was approximately 1.2 V at 25 °C, which was similar to that obtained by a study of an analytical model of a DMFC where the standard thermodynamic/reversible potential was equal to 1.214 V [34]. As evident in Figure 10, as the Gibbs free energy increases, the theoretical voltage increases, which is expected. On the other hand, in Figure 9, an inverse relationship is exhibited between the voltage and temperature of the fuel cell. In other words, as the temperature increases, the reversible cell voltage linearly decreases. This decrease can be justified by Equation (9), where an increase in the temperature is associated with an increase in the $T∆\bar{S}$ term, which consequently decreases the molar Gibbs free energy change in formation, thus decreasing the reversible cell voltage.

**Figure 11:**
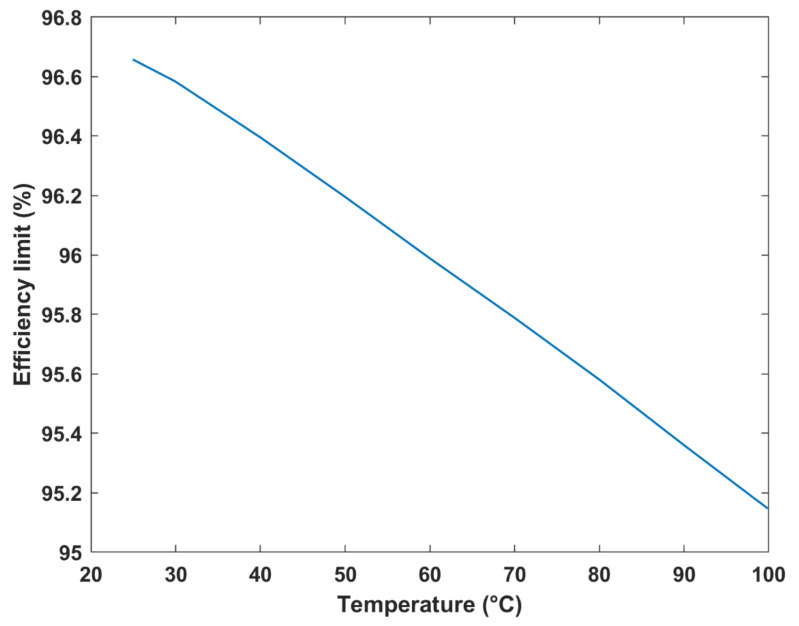
Maximum efficiency vs. temperature.

The maximum efficiency of the DMFC system was investigated at different operating temperatures, as shown in Figure 11. Evidently, the cell can attain a maximum efficiency limit of 96.6% and a minimum of 95.2%, thus exhibiting a 1.45% decrease in efficiency upon increasing the temperature by around 75 °C. In general, a negative correlation exists between the efficiency limits of the fuel cell and temperature. The decrease in efficiency with temperature is attributed to the decrease in Gibbs free energy with an increasing temperature.

**Figure 12:**
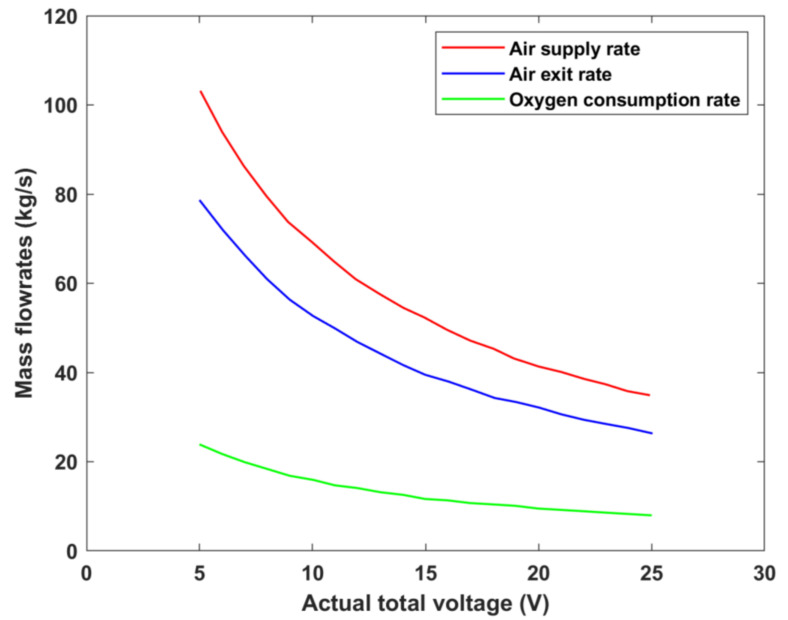
Variation in oxygen consumption rates and air flowrates with operating cell voltage.

**Figure 13:**
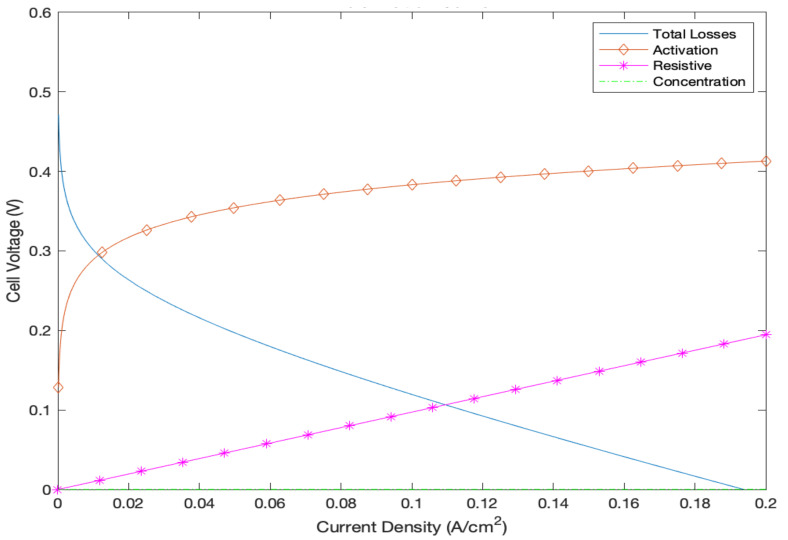
Polarization curve with loss breakdown.

The variation in air inlet and air exit flowrates, as well as the consumption rates of oxygen, can be investigated by varying the cell’s operating voltage, as shown in Figure 12. It is evident that the exit air’s mass flowrate is lower than that supplied owing to the oxygen being consumed during the electrochemical reaction taking place within the cell [35]. In addition, the irreversibilities of the DMFC system were also investigated by computing the polarization curves in order to obtain a more accurate representation of the output cell voltage, as evident in Figure 13. The figure demonstrates the voltage drops occurring due to activation, ohmic, and concentration losses as a function of the current density. As is evident, the concentration losses are almost nonexistent. This is justified by the fact that the current density range under investigation is a minute (only up to 0.2 A/cm^2^). Generally, concentration losses are usually more dominant under higher current densities [6]. The figure also makes it evident that activation losses are the most dominant form of irreversibilities owing to the high methanol crossover issue that commonly appears within DMFC systems. In addition, when the fuel cell is operated at maximum current density (0.2 A/cm^2^), the output cell voltage is almost 0 V owing to the fact that the irreversibilities are highest at this operating current density.

**Figure 14:**
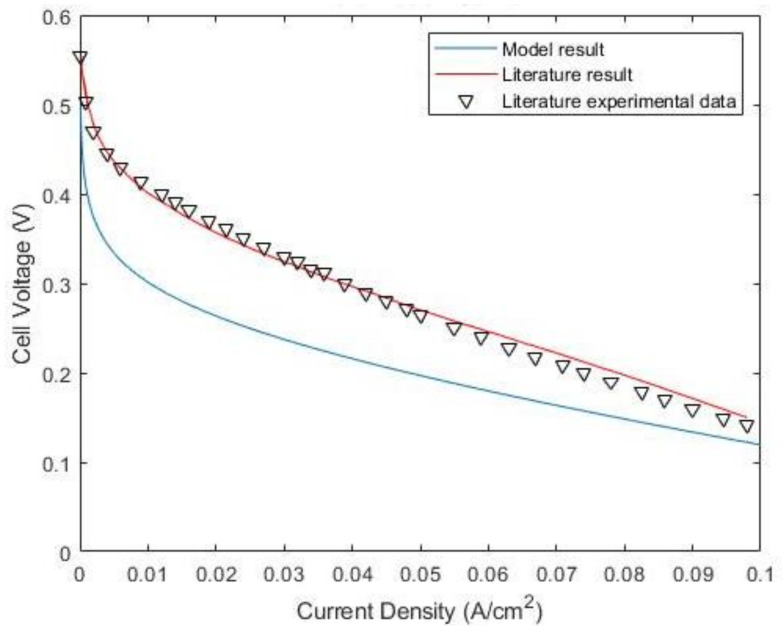
Polarization curve by Shrivastava et al. [36] (red line and black triangles) and the polarization curve obtained from MATLAB (blue line).

To ensure the results conform to the results in the literature, the actual output cell voltage, which takes into account the aforementioned irreversibilities, was validated against the output cell voltage curve from another study, which modeled a passive DMFC to evaluate its performance [36], as shown in Figure 14. As shown in the figure, the overall profile of the obtained curve coincides with what is available in the literature. In general, it can be concluded that the computed output cell voltage curve can serve as an adequate representation of the DMFC.

**Figure 15:**
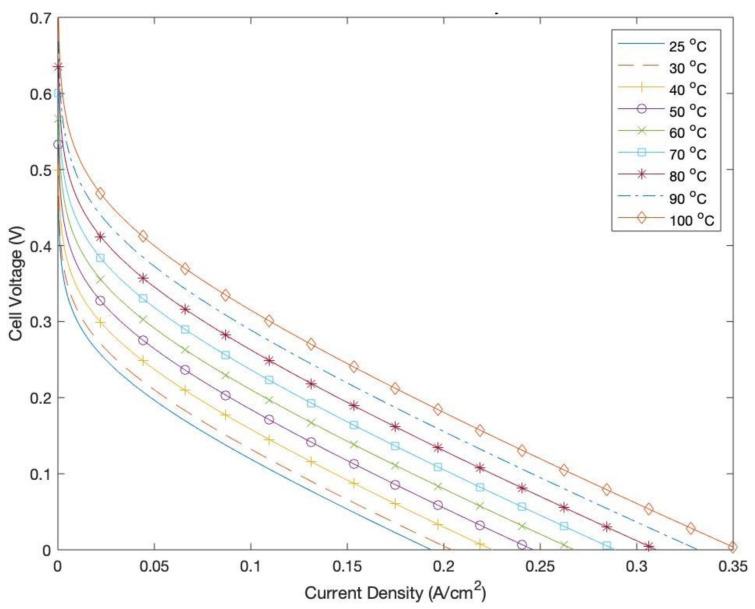
Polarization curve at various temperatures.

Figure 15 highlights the effect of increasing the operating temperature of the DMFC on its output cell voltage. It is clear that increasing the temperature increases the actual cell voltage. This is owed to the enhanced kinetics of the MOR [37]. In addition, at higher temperatures, the electrodes of the DMFC are more reactive, which results in higher exchange current densities and lower activation overpotentials, thus enhancing the output voltage of the cell. Despite the enhanced performances attributed to increased operating temperatures, it is always imperative to ensure that the temperature does not increase beyond certain critical values, as that can possibly deteriorate the cell performance by causing the cells to dry up and suffer from insufficient humidity [38].

## 7. Conclusions

This study provides a concise overview of DMFCs and investigates the performance of a DMFC used for military applications known as JENNY 600S. The analysis included computing the basic performance characteristics of the fuel cell, such as the gravimetric and volumetric power densities of the cell; Gibbs free energy at various different operating temperatures; and its correlation with various other parameters. Moreover, the theoretical reversible voltage was calculated, its relationship with temperature was investigated, and an inverse relationship was evident. The results showed that as the temperature increases the theoretical voltage decreases owing to the decrease in Gibbs free energy with the increasing temperature. Furthermore, the efficiency limit of the fuel cell was examined with respect to temperature, and it was confirmed that the efficiency limits of the DMFC drop with increasing temperature. In addition, the inlet and exit flowrates of air and oxygen were obtained for the examined fuel cell. The results showed that the exit mass flowrate of air was always lower than that supplied due to the consumption of oxygen during electrochemical reactions. Finally, the polarization curve was plotted to examine the various overpotentials independently and their effect on the output cell voltage at different temperatures. It was evident that increasing the temperature of the fuel cell enhanced the output voltage owing to enhanced methanol oxidation reactions and lower activation overpotentials, which also resulted in the fuel cell operating at increased current densities.

## Figures and Tables

**Table 1:** Common types of fuel cells.

Fuel Cell	Abbreviation
Alkaline Fuel Cell	AFC
Direct Methanol Fuel Cell	DMFC
Phosphoric Acid Fuel Cell	PAFC
Polymer Electrolyte Membrane Fuel Cell	PEMFC
Solid Oxide Fuel Cell	SOFC
Molten Carbonate Fuel Cell	MCFC

**Table 2:** Comparison of catalyst performance according to different studies [20].

Catalyst	Electrochemical Surface Area (ECSA) (m^2^ g^−1^)	Mass Activity (mA mg_pt_^−1^)	Specific Activity (mA cm^−2^)	CO Oxidation Peak Potentials
Pt/CeO_2_ NW ^1^	30.2	438.3	1.45	−0.005 V versus Hg Hg_2_SO_4_
PtCu NWN ^2^	82	1287	1.87	0.488 V versus SCE ^3^
PtRu nanosponge	48.4	410	-	0.251 V versus SCE
PtRuCuW	26	467.1	1.8	0.47 V versus SCE
Pt-Cu/GN-CD ^4^	44.6	1360	-	0.57 V versus Ag/AgCl
Pt-Ni/GN-CD	26.5	960	-	0.58 V versus Ag/Agcl
Pt-Co/GN-CD	30.2	840	-	0.60 V versus Ag/Agcl
Pt/GN-CD	20.4	500	-	0.61 V versus Ag/Agcl
FePtSn/C	40.7	1077	1.27	0.45 V versus SCE
PtRu/N-GA ^5^	-	668	-	0.44 V versus Ag/AgCl
PtFe/C	-	575	1.6	0.53 V versus SCE

^1^ NW—nanowires. ^2^ NWN—nanowire networks. ^3^ SCE—saturated calomel electrode. ^4^ GN-CD—β-cyclodextrin-functionalized graphene nanosheets (GNs-CD). ^5^ N-GA—N-doped graphene aerogel.

**Table 3:** DMFC compositions and operating conditions of various groups [25].

Group	Type/Electrolyte	Catalyst		Loading mg/cm^2^	Temperature °C	Anode Feed	Cathode Feed	Pressure		Cell Performance at 400 mA/cm^2^
		Anode	Cathode					Anode	Cathode	
Siemens	Vapor-feed Nafion-117	Pt/Ru	Pt	8.0 Pt	130	2 M	O_2_	4.4 bar	5.0 bar	0.52 V
Newcastle	Vapor-feed Nafion-117	Pt/Ru/C	Pt/C	2.5 Pt	98	2 M	O_2_ air	Ambient Ambient	5.0 bar 5.0 bar	0.5 V 0.4 V
LANL	Liquid-feed Nafion-112	PtRuO_x_	Pt black	2.2, 2.3 Pt	130 110	1 M	O_2_ air	3 atm 1.8 atm	5.0 bar 3.0 bar	0.57 V 0.52 V
LANL	Liquid-feed Nafion-117	PtRuO_x_	Pt black	2.2, 2.3 Pt	130 110	1 M	O_2_ air	3 atm 1.8 atm	5.0 bar 3.0 bar	0.47 V 0.39 V
JPL Giner	Liquid-feed Nafion-117	Pt/Ru/C	Pt/Ru/C	4	90 90	1 M 1 M	O_2_ air	Ambient Ambient	2.36 atm 2.36 atm	0.47 V 0.38 V

**Table 4:** Technical data of the JENNY 600S DMFC.

Technical Data	
Deployment elevation	Up to 4000 m
Noise emission	<37 dB(A) at 1 m
Charging performance per day	600 Wh
Nominal power	25 W
Output voltage	10–30 V DC
Weight	1.7 kg
Dimensions (L × W × H)	184 × 74 × 252 mm

## Data Availability

All data were mentioned in this paper. No external supporting data are available.

## Nomenclature

$P_{e}$	Power (W)
*F*	Faraday Number
*V_c_*	Actual Voltage (V)
λ	Stoichiometry
*A*	Surface Area (m^2^)
$E_{oc}$	Open Circuit Voltage (V)
$E_{activation}$	Activation Voltage Losses (V)
$E_{ohmic}$	Ohmic Voltage Losses (V)
$E_{concentration}$	Concentration Voltage Losses (V)
$E_{anode}$	Anode Voltage Losses (V)
$E_{cathode}$	Cathode Voltage Losses (V)
*I*	Current (A)
*i*	Current Density (A cm^−2^)
*n*	Number of Moles
${\bar{g}}_{f}$	Gibbs Free Energy of Formation (kJ mol^−1^)
${\bar{h}}_{f}$	Enthalpy of Formation (kJ mol^−1^)
η	Efficiency
$\bar{S}$	Molar Entropy (J mol^−1^ K^−1^)

## References

[1] Roser M., Ritchie H., Ortiz-Ospina E. World Population Growth. https://ourworldindata.org/world-population-growth.

[2] Ritchie H., Roser M. Fossil Fuels. https://ourworldindata.org/fossil-fuels.

[3] Sun C., Negro E., Vezzù K., Pagot G., Cavinato G., Nale A., Bang Y.H., Di Noto V. (2019). Hybrid inorganic-organic proton-conducting-conducting membranes based on SPEEK doped with WO_3_ nanoparticles for application in vanadium redox flow batteries. J. Electrochim. Acta.

[4] Sun C., Negro E., Nale A., Pagot G., Vezzu K., Zawodzinski T.A., Meda L., Gambaro C., Di Noto V. (2021). An efficient barrier toward vanadium crossover in redox flow batteries: The bilayer [Nafion/(WO_3_)_x_] hybrid inorganic-organic membrane. J. Electrochim. Acta.

[5] Bellis M. Hydrogen Fuel Cells Innovation for the 21st Century. https://www.thoughtco.com/hydrogen-fuel-cells-1991799.

[6] Sharaf O.Z., Orhan M.F. (2014). An overview of fuel cell technology: Fundamentals and applications. J. Renew. Sustain. Energy Rev..

[7] Zhang H., Sun C. (2021). Cost-effective iron-based aqueous redox flow batteries for large-scale energy storage application: A review. J. Power Sources.

[8] Hamada A.T., Orhan M.F. (2022). An overview of regenerative braking systems. J. Energy Storage.

[9] Lv F., Wang Z., Shi L., Zhu J., Edström K., Mindemark J., Yuan S. (2019). Challenges and development of composite solid-state electrolytes for high-performance lithium ion batteries. J. Power Sci..

[10] Letsau T.T., Govender P.P., Msomi P.F. (2022). Imidazolium-Quaternized Poly(2,6-Dimethyl-1,4-Phenylene Oxide)/Zeolitic Imidazole Framework-8 Composite Membrane as Polymer Electrolyte for Fuel-Cell Application. Polymers.

[11] Bodrick C., Lipman T.E., Farshchi M., Lutsey N.P., Dwyer H.A., Sperling D., Gouse S.W., Harris D.B., King F.G. (2002). Evaluation of fuel cell auxiliary power units for heavy-duty diesel trucks. Transp. Res. Part D Transp. Environ..

[12] Cowey K., Green K.J., Mepsted G.O., Reeve R. (2004). Portable and military fuel cells. Curr. Opin. Solid State Mater. Sci..

[13] Patil A.S., Dubois T.G., Sifer N., Bostic E., Gardner K., Quah M., Bolton C. (2004). Portable fuel cell systems for America’s army: Technology transition to the field. J. Power Sources.

[14] Li X., Faghri A. (2013). Review and advances of direct methanol fuel cells (DMFCs) part I: Design, fabrication, and testing with high concentration methanol solutions. J. Power Sources.

[15] Nakagawa N., Sekimoto K., Masdar M.S., Noda R. (2009). Reaction analysis of a direct methanol fuel cell employing a porous carbon plate operated at high methanol concentrations. J. Power Sources.

[16] Sone Y., Ekdunge P., Simonsson D. (1996). Proton conductivity of Nafion 117 as measured by a four-electrode AC impedance method. J. Electrochem. Soc..

[17] Hoppe W., Thonemann N., Bringezu S. (2018). Life cycle assessment of carbon dioxide-based production of methane and methanol and derived polymers. J. Ind. Ecol..

[18] Zhang H., Chen N., Sun C., Luo X. (2020). Investigations on physicochemical properties and electrochemical performance of graphite felt and carbon felt for iron-chromium redox flow battery. Int. J. Energy Res..

[19] Spiegel C. Direct Methanol Fuel Cell Improvements. Fuel Cell Store. https://www.fuelcellstore.com/blog-section/direct-methanol-fuel-cell-improvements.

[20] Gong L., Yang Z., Li K., Xing W., Liu C., Ge J. (2018). Recent development of methanol electrooxidation catalysts for direct methanol fuel cell. J. Energy Chem..

[21] Mo J., Kang Z., Retterer S.T., Cullen D.A., Toops T.J., Green J.B., Mench M.M., Zhang F.Y. (2016). Discovery of true electrochemical reactions for ultrahigh catalyst mass activity in water splitting. Sci. Adv..

[22] Arico A.S., Baglio V., Antonucci V. (2010). Direct Methanol Fuel Cells.

[23] Igwe C., Achebe C., Chinweze A. (2022). Review of alternative energy production methods by oxidation of electrolyzed hydrogen in a hydrogen cell. Glob. Sci. J..

[24] Hibbs M., Hickner M.A., Alam T.M., McIntyre S.K., Fujimoto C.H., Cornelius C.J. (2008). Transport properties of hydroxide and proton conducting membranes. Chem. Mater..

[25] Hogarth M.P., Hards G.A. (1996). Direct Methanol Fuel Cells. Johns. Matthey Technol. Rev..

[26] Gouérec P., Poletto L., Denizot J., Sanchez-Cortezon E., Miners J.H. (2004). The evolution of the performance of alkaline fuel cells with circulating electrolyte. J. Power Source.

[27] Chiu Y.J., Yu T.L., Chung Y.C. (2011). A semi-empirical model for efficiency evaluation of a direct methanol fuel cell. J. Power Sources.

[28] Majidi P., Altarawneh R.M., Ryan N.D.W., Pickup P.G. (2016). Determination of the efficiency of methanol oxidation in a direct methanol fuel cell. Electrochim. Acta.

[29] Liu P., Yin G.-P., Du C. (2008). Composite anode catalyst layer for direct methanol fuel cell. Electrochem. Commun..

[30] Deodath R., Jhingoorie J., Riverol C. (2017). Direct methanol fuel cell system reliability analysis. Int. J. Hydrogen Energy.

[31] Bahrami H., Faghri A. (2011). Exergy analysis of a passive direct methanol fuel cell. J. Power Sources.

[32] Deng H., Jiao D., Zu M., Chen J., Jiao K., Huang X. (2015). Modeling of passive alkaline membrane direct methanol fuel cell. Electrochim. Acta.

[33] Garcia-Diaz B., Patterson J., Weidner J. (2012). Quantifying Individual Losses in a Direct Methanol Fuel Cell. J. Fuel Cell Sci. Technol..

[34] Rosenthal N., Vilekar S., Datta R. (2012). A comprehensive yet comprehensible analytical model for the direct methanol fuel cell. J. Power Sources.

[35] Hawkes G., O’Brien J.E., Haberman B.A., Marquis A.J., Baca C.M., Tripepi D., Costamagna P. Numerical Prediction of the Performance of Integrated Planar Solid-Oxide Fuel Cells, with Comparisons of Results from Several Codes. Proceedings of the International Conference on Fuel Cell Science, Engineering and Technology.

[36] Shrivastava N., Thombreb S., Wasewar K. (2012). Modelling of Passive Direct Methanol Fuel Cell for Performance Evaluation. i-Manag. J. Future Eng. Technol..

[37] Cheng Y., Zhang J., Lu S., Jiang S.P. (2020). Significantly enhanced performance of direct methanol fuel cells at elevated temperatures. J. Power Sources.

[38] Govindarasu R., Somasundaram S. (2020). Studies on Influence of Cell Temperature in Direct Methanol Fuel Cell Operation. Processes.

